# The Use of Immunohistochemistry, Fluorescence *in situ* Hybridization, and Emerging Epigenetic Markers in the Diagnosis of Malignant Pleural Mesothelioma (MPM): A Review

**DOI:** 10.3389/fonc.2020.01742

**Published:** 2020-09-09

**Authors:** Eric Rozitis, Ben Johnson, Yuen Yee Cheng, Kenneth Lee

**Affiliations:** ^1^Sydney Medical School, The University of Sydney, Sydney, NSW, Australia; ^2^Asbestos Diseases Research Institute, Concord, NSW, Australia; ^3^Anatomical Pathology Department, NSW Health Pathology, Concord Repatriation General Hospital, Concord, NSW, Australia

**Keywords:** malignant pleural mesothelioma, CirRNA, DNA methylation, microRNA, epigenetic biomarkers, biomarkers

## Abstract

Malignant pleural mesothelioma (MPM) is an aggressive asbestos related disease that is generally considered to be difficult to diagnose, stage and treat. The diagnostic process is continuing to evolve and requires highly skilled pathology input, and generally an extensive list of biomarkers for definitive diagnosis. Diagnosis of MPM requires histological evidence of invasion by malignant mesothelial cells often confirmed by various immunohistochemical biomarkers in order to separate it from pleural metastatic carcinoma. Often when invasion of neoplastic mesothelial cells into adjacent tissue is not apparent, further immunohistochemical testing - namely BAP1 and MTAP, as well as FISH testing for loss of p16 (CDKN2A) are used to separate reactive mesothelial proliferation due to benign processes, from MPM. Various combinations of these markers, such as BAP1 and/or MTAP immunohistochemistry alongside FISH testing for loss of p16, have shown excellent sensitivity and specificity in the diagnosis of MPM. Additionally, over the recent years, research into epigenetic marker use in the diagnosis of MPM has gained momentum. Although still in their research stages, various markers in DNA methylation, long non-coding RNA, micro RNA, circular RNA, and histone modifications have all been found to support diagnosis of MPM with generally good sensitivity and specificity. Many of these studies are however, limited by small sample sizes or other study limitations and further research into the area would be beneficial. Epigenetic markers show promise for use in the future to facilitate the diagnosis of MPM.

## Introduction

Malignant pleural mesothelioma (MPM) is an aggressive malignancy which arises from the serosal lining of the pleural surface and is primarily caused by the prior inhalation of asbestos particles (1). A peritoneal form, also referred to as peritoneal mesothelioma is considered to be caused by the ingestion of asbestos particles. Diagnosis of MPM continues to evolve and grow as novel molecular and antibody testing options become available. Whilst histology with immunohistochemistry (IHC) remains the cornerstone and gold standard technique for the clinical diagnosis of MPM, emerging molecular techniques have led to the discovery of novel MPM-specific biomarkers, which are becoming increasingly utilized in conjunction with IHC to obtain a definitive diagnosis (2).

MPM, and all its subtypes, is generally considered to be an occupational disease, with more than 80% of cases being due environmental exposure to asbestos particles (3). The diagnosis of suspected MPM aside from a thorough occupational history, physical examination, relevant general laboratory blood work, computer tomography of the chest and abdomen; should also include at least a cytology specimen, but in most cases, histopathological diagnosis is required via thoracoscopic biopsy (3). Approximately 80–90% of mesotheliomas arise from the pleura, with the remainder being from the peritoneum (4). The separation of MPM from reactive mesothelial hyperplasia (RMH) has been a particularly challenging area.

Advances in both immunohistochemical markers as well as fluorescence *in situ* hybridization and epigenetic markers have proven helpful. These ancillary tests can help separate florid RMH from MPM in equivocal cases (5). Immunohistochemistry (IHC), fluorescence *in situ* hybridization (FISH), and various other molecular markers (including epigenetic markers) have sufficient evidence behind their use however, some are still in their research stages, or yet to be used widely in clinical diagnosis. Recent studies showing homozygous loss of p16 (CDKN2A) by FISH, losses of BRCA-1 associated protein-1 (BAP1) and methylthioadenosine phosphorylase (MTAP) by IHC as being very specific for MPM (6–14). MTAP gene loss is usually co-deleted with p16 gene (8), and MTAP IHC loss is a good surrogate for p16 gene deletion, which is an additional useful option in current histopathological diagnosis (12). Conversely, epigenetic markers such as DNA methylation profiles and micro RNA and circular RNA dysregulation, although supported by promising literature, are not used in clinical practice currently (2, 6). Lastly, epigenetic markers such as methylation markers, long non-coding RNAs, miRNAs, and circular RNAs have emerged in the literature over the years and although not yet incorporated into clinical practice, trials have shown promise in their potential use in MPM diagnosis. MPM is a devastating disease once identified, and the need for reliable, efficient, and cost-effective diagnostics is essential. The above mentioned diagnostic principles and molecular markers will be explored in more detail throughout this paper.

### Aspects of Diagnosis

#### Cytology of Malignant Pleural Mesothelioma

MPM is classically difficult to diagnose on a cytological basis. Because mesotheliomas classically present with serous pleural effusion, these specimens are often submitted for evaluation. Sensitivity for cytological diagnosis is thought to range from 30 to 75%, with a higher false negative rate likely being due to sampling error rather than interpretation (15). Many cytologic features of MPM are shared with reactive mesothelial proliferation, such as high nucleus-to-cytoplasm ratio and scalloped cell clump borders. Cytological examination also often precludes the pathologist from examining pre-existing surrounding tissue which is often one of the crucial diagnostic features of MPM. Another hindrance of cytology in the diagnosis of sarcomatoid MPM is that malignant cells are generally not shed into the effusion, which may hamper diagnostic value of the cytology specimen (16). Therefore, sarcomatoid mesothelioma is rarely diagnosed on effusion cytology. Literature continues to emerge favoring the notion that positive stains for p53, epithelial membrane antigen, IMP-3, glucose transporter-1 and CD146 can differentiate between malignant and benign reactive mesothelial cells, however, it is accepted now that these markers should not be used to differentiate individual cases (2). The difficulty in diagnosing MPM in cytology specimens have been a major issue particularly for pathologist until recently with the advent of BAP-1 and MTAP IHC, and loss of p16 by FISH (11).

#### Histologic Overview of Malignant Pleural Mesothelioma

There are three subtypes of MPM and the histologic features are summarized in Table 1. In terms of histological examination, the definitive diagnosis of MPM is characterized by underlying tissue invasion by malignant mesothelial cells of which can be highlighted by the mesothelial markers discussed in Table 2. Whereas this feature of tissue invasion is absent in RMH (3). As previously noted, it is important for the pathologist to beware of fibrotic entrapment of mesothelial cells. MPM features a dense cellularity whereas in mesothelial hyperplasia, although cellularity is often prominent, the cells are confined to mesothelial surface or pleural space (2, 3). Reactive mesothelium may display simple papillae but they are lined by a single cell layer of mesothelium. Necrosis may be present in MPM, however, empyema may also display such a feature and thus this feature is not diagnostic of MPM. MPM often tends to feature a disorganized growth pattern (especially on cytokeratin staining) but uniform growth is usually seen in the reactive mesothelial proliferations. Degree of mitotic activity and cytologic atypia are not helpful in the differentiation of these two entities (2, 12). The epithelioid form is the most common with the best prognosis, and sarcomatoid subtype being the least common and with it carries the least favorable prognosis (2).

**TABLE 1:** Malignant pleural mesothelioma subtype and the associated histologic features (2).

Mesothelioma subtype	Histolopathologic features
Epithelioid	Polygonal, oval, or cuboidal cells arranged in various growth patterns including papillary, tubular/acinar (glandular), tubulopapillary, adenomatoid, deciduoid, solid. Psammoma bodies and necrosis may be present.
Sarcomatoid	Pleomorphic spindled cells arranged in fascicles, whorls or in a haphazard fashion. Heterologous elements (cartilage or bone) may be present. Pleomorphic spindled cells may be entrapped in a dense sclerotic stroma (desmoplastic variant).
Biphasic	Combination of epithelioid and sarcomatoid features.

**TABLE 2:** Initial limited panel to differentiate cells of mesothelial origin from adenocarcinoma (epithelial)* (2, 18, 19).

Recommended mesothelial markers (at least two)	Recommended adenocarcinoma (epithelial) markers, at least two
Calretinin	CEA monoclonal
CK 5/6	BerEP4
WT-1	MOC-31
D2-40	Claudin-4
	B72.3

* Not comprehensive list.

#### Differentiating Mesothelioma From Metastatic Carcinomas

There exists a wide variety of immunohistochemical markers which can be used to separate metastatic carcinomas from reactive mesothelial cells and from primary pleural neoplasms. Light microscopy is an essential primary modality in assessing tissue morphological changes and is generally used alongside various immunohistochemical marker panels to differentiate between these entities (2). In regard to immunohistochemical diagnosis, the general consensus is that at least two carcinoma and two mesothelial markers should be used for the diagnosis of MPM. This has been recommended by The International Mesothelioma Interest Group (2).

Primary pleural neoplasms, such as MPM, are relatively rare entities when compared with the much higher frequency of metastatic carcinomas to the pleura. Carcinoma diagnoses vastly outnumber MPM diagnoses. Fortunately there are a number of highly applicable and effective immunohistochemical (IHC) markers which serve to differentiate between these two malignancies (2). These IHC markers often serve to complement tumor morphology which is appreciated on histopathological examination.

Primary pleural masses include benign tumors, tumors with low malignant potential, and malignant tumors. Differentiation between these forms often relies heavily on morphology, where the malignant tumors often feature deep stromal invasion, complex growth patterns, and dense cellularity (17). The latter two features can also be present in benign pleural growths, however, it is the stromal invasion which is generally the indicator of malignancy. Benign pleural tumors are beyond the scope of this paper and will not be discussed here.

**FIGURE 1:**
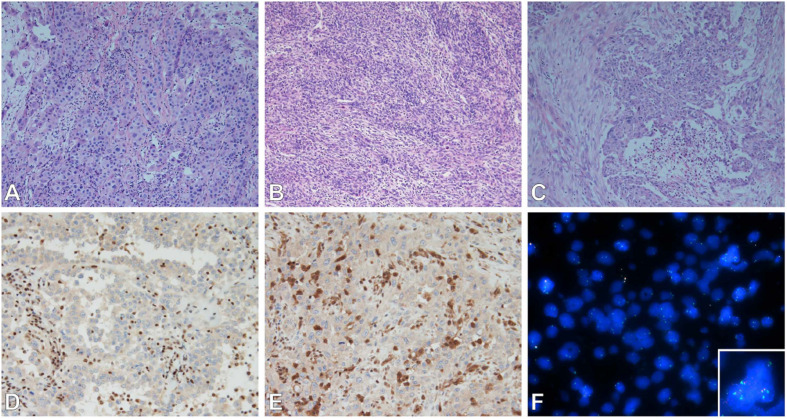
**(A–C)** Subtypes of mesothelioma. **(A)** Epithelioid, **(B)** sarcomatoid, **(C)** biphasic. Epithelioid subtype with BAP-1 **(D)**, MTAP **(E)** immunohistochemistry (loss of nuclear and cytoplasmic staining respectively); and FISH **(F)** of MTAP/CDKN2A/CEP9 tricolor probes (MTAP – Aqua, CDKN2A – Red, CEP9 – Green) showing loss of MTAP and CDKN2A (inset normal signals for comparison). Images **(A–E)** were taken by Olympus microscope with 20× objective, image **(F)** was taken by ZEISS Axio.M2 microscope with 63× objective.

Epithelioid MPM needs to be differentiated from RMH and metastatic carcinomas to the pleura. Representative Haemotoxylin and Eosin staining of three mesothelioma subtypes are shown in Figures 1A–C. Calretinin, cytokeratin 5 or 5/6, WT1, and podoplanin (D2-40), are generally positive in mesothelial cells, and are the best markers for epithelioid mesothelioma (2, 18). Metastatic carcinomas are diagnosed from their clinical presentation and their characteristic histological appearance. They retain their own characteristic immunophenotype. Pulmonary adenocarcinoma generally stains positive for claudin-4, TTF-1, MOC31, CEA, B72.3, BG8, and BER-EP4 markers, with TTF-1 and napsin-A having the highest specificity lung adenocarcinoma (19). When differentiating between epithelioid MPM and squamous cell carcinoma, the latter tends to stain positive for p40, p63 (less useful as it cross reacts with adenocarcinoma). Table 2 outlines the primary IHC markers which can be initially used in the differentiation between mesothelial cells and carcinoma (epithelial) cells.

The important differentials of sarcomatoid MPM include other malignant spindle cell neoplasms such as synovial sarcoma (SS), malignant solitary fibrous tumur (SFT), sarcomatoid carcinoma, malignant peripheral nerve sheath tumour (MPNST), and metastatic spindle cell melanoma. To support the diagnosis of sarcomatoid MPM, immunohistochemical markers such podoplanin (D2-40) and pancytokeratin may be used as they can be expressed in sarcomatoid mesotheliomas, but more specific mesothelial markers such as calretinin may only be expressed in up to 30% of sarcomatoid MPM (2). The distinction between sarcomatoid MPM and pulmonary sarcomatoid carcinoma may be extremely difficult histopathologically at times, and in such instances requires close clinical and radiological correlation.

In regards to breast cancer, metastatic infiltrating carcinoma of the breast tends to be CK7, ER, PR, GCDFP positive, but negative for TTF-1, and calretinin. GATA3 is often positive in breast cancers as well, but a proportion of epithelioid mesotheliomas also express it (20).

In regard to metastatic renal cell carcinoma and ovarian carcinoma, PAX8 tends to be positive for these two malignancies (21). However, it should be noted that PAX8 may also sometimes be positive for benign peritoneal mesothelium and peritoneal mesotheliomas, and caution should be exercised when using this marker to differentiate ovarian serous tumors from peritoneal mesothelial proliferations, both benign and malignant (22).

**FIGURE 2:**
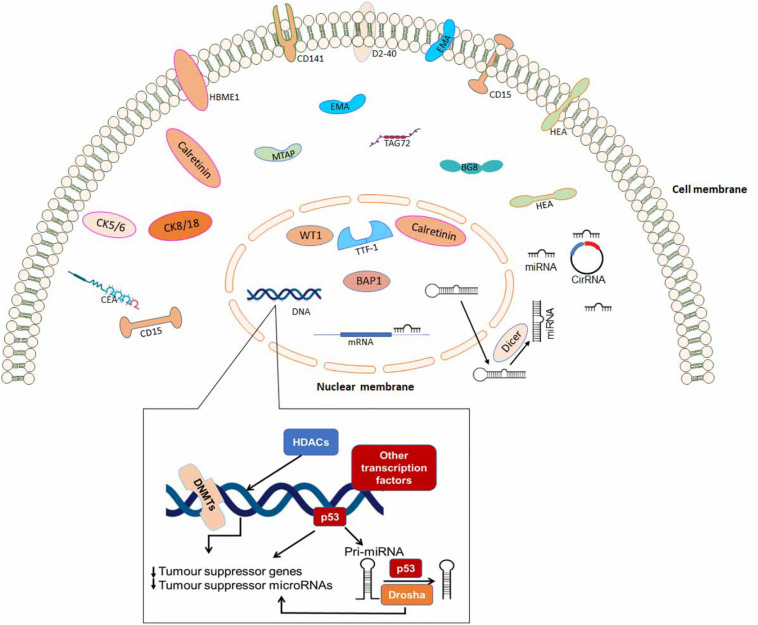
A schematic diagram indicating protein localization for immunohistochemistry staining and epigenetic biomarkers in mesothelioma cell.

Some of the immunohistochemical markers which can be used to differentiate between various metastatic carcinomas are outlined in Table 3. Finally, Figure 2 is a schematic diagram indicating protein localization for IHC staining as well as other epigenetic biomarkers in mesothelioma cell.

**TABLE 3:** Additional immunohistochemical markers to aid in determining origin of metastatic adenocarcinoma or metastatic squamous cell carcinoma (2, 18, 19).

Organ specific carcinoma	Additional immunohistochemical markers
Lung adenocarcinoma	TTF-1, Napsin-A
Breast carcinoma	GATA-3, ER, PR, mammoglobin
Colon adenocarcinoma	CK20, CDX-2
Gynecological tract adenocarcinoma	PAX-8, ER
Squamous cell carcinoma	P63, P40

Biphasic tumors often comprise elements of both epithelioid and sarcomatoid tumors (2). The mesothelial markers discussed as above applies to this subtype as well (23).

#### Differentiating Malignant Pleural Mesothelioma (MPM) From Reactive Mesothelial Hyperplasia (RMH)

RMH is well known for mimicking MPM because the former entity can often demonstrate typical classic signs associated with malignancy (2). Separating MPM from reactive mesothelium is obviously an important differentiation to make as the former carries a poor prognosis while the latter generally requires no further treatment. Tissue invasion, although being a classic sign of MPM, can also be imitated by a reactive mesothelial proliferation in that mesothelial cells can be entrapped within areas of fibrosis which can often be seen as consistent with an invasive process. This is not helped by the fact that invasion in MPM is often subtle, can lack a desmoplastic reaction, and malignant cells may only extend from the mesothelial space into just a few layers of collagenous tissue. Invasion can be highlighted by the use of stains such as pancytokeratin and calretinin to highlight the presence of mesothelial cells in the underlying stroma, fat or skeletal muscle. If a cellular proliferation has been established as mesothelial in origin, the presence of true invasion is considered to support a definite diagnosis of MPM (2). With regard to the separation of reactive mesothelium from true MPM, traditional immunohistochemical markers used in the past have proven to not very useful. Markers such as p53, EMA, desmin, insulin-like growth factor 2, IMP-3, GLUT-1 have not classically been beneficial, whereas loss of nuclear and cytoplasmic BAP1 and MTAP staining on IHC (Figures 1D,E) respectively, and p16 deletion by FISH (Figure 1F) generally have a higher specificity in addition to good sensitivity (6, 13, 24). Both of these will be discussed in further detail below. The sensitivities and specificities of the currently researched IHC markers and p16 FISH when used in the diagnosis of MPM are detailed in Table 4.

**TABLE 4:** Sensitivity and specificity of individual and combined immunohistochemical markers and p16 loss by FISH.

	BAP-1 IHC	MTAP IHC	BAP-1 and MTAP IHC	BAP-1 and p16 loss FISH
Sensitivity	36.7−67.0%(11, 13, 37)	42.2−74.2%(7, 11, 12)	76.5−90.0%(5, 7, 11, 14)	80.0−100%(7, 13, 27, 29, 37)
Specificity	100%(11, 13, 37)	100%(11, 12)	100%(5, 7, 11, 14)	100%(13, 27, 29, 37, 38)

### Loss of BAP1, p16, MTAP and NF2

#### Immunohistochemical Diagnosis – Loss of BAP-1

Some of the main molecular alterations in the development of MPM have been known for many years, however, their implication in diagnosis continues to evolve (13). The most commonly referred to markers in malignant mesothelioma are loss of BAP1 by IHC, and the deletion of p16 by FISH.

BAP1 is considered the most common acquired and germline mutation in MPM. It has a role in cellular proliferation and growth inhibition, and decreased levels leads to genomic instability which subsequently increases the risk of neoplastic transformation (6). The BAP-1 gene is located on the short arm of chromosome 3 (3p21) (25). When BAP-1 IHC is used to differentiate between MPM and reactive mesothelium, the sensitivity has been found to range from 61 to 67% with a specificity of 100%. Therefore, BAP1 is considered to be highly specific for discriminating MPM from RMH (13, 24). One drawback of the use of BAP1 IHC is that BAP1 mutation and thus BAP1 protein loss may occur in other metastatic pleural malignancies, such as lung, breast, melanoma, and kidney tumors (25, 26).

One of the complicating factors of use BAP-1 IHC is that not all MPM and reactive mesothelial proliferation cases have homogenous staining. Thus, it can occasionally be difficult to determine a positive or negative result. Cut-off values for what contributes a positive stain have also not yet been established (13). The involvement of, and relationships between, groups of cells showing p16 and BAP-1 expression in the diagnosis of MPM have not yet been defined (27). This is however, unfortunately complicated by the fact that the loss of BAP1 is uncommon in the sarcomatoid and desmoplastic forms of mesothelioma (24, 28). Another issue is that most mesotheliomas of peritoneal origin do show a loss of BAP1 by IHC but do not show loss of p16 by FISH. Nevertheless, BAP-1 IHC is currently being used as part of the diagnostic armamentarium for MPM.

Combined use of BAP-1 IHC and p16 FISH (the latter of which will discussed in more detail in a later section) is highly useful. In several studies, the loss of BAP1 by IHC and the homozygous deletion of p16 by FISH is very specific for mesothelioma but still may lack sensitivity to a certain extent. Specificity was 100% in three recent studies, with their associated sensitivities 58% (29), 93% (13), and 100% (27). One limitation of most of these studies is that they tend to examine nuclear expression of the BAP1 protein rather than assessing actual BAP1 gene alteration. Although studies have found excellent concordance between BAP1 gene dysfunction and protein expression, a measured loss of BAP1 nuclear staining may not always be due to a BAP1 genetic alteration in every case (6, 30).

#### The FISH Assay – Loss of p16, MTAP, and NF2 Markers

The advantage of the FISH assay when compared to conventional PCR is its ability to identify homozygous and hemizygous deletions in areas of tumor tissue which can be visualized and examined at the same time. The FISH assay can be performed using a typical dual-color FISH probe on paraffin-embedded tissue and is relatively less expensive than other molecular assays. Molecular techniques are generally slower, more expensive, and have a longer turnaround time when compared to the FISH assay. The homozygous deletion of the 9p21 locus involves a cluster of genes such as p16, CDKN2B, and MTAP (8, 31). In particular for 9p21 deletions, FISH has been shown by multiple studies to be a highly useful technique (9, 10, 19). Some of the major sensitivities and specificities of various combinations of IHC and FISH markers are outlined in Table 4.

Using FISH to identify a p16 deletion is currently used in clinical practice and is considered a highly useful adjunct to IHC (8). A homozygous deletion is diagnostic of malignancy in a mesothelial proliferation, however, p16 deletion is not considered useful in differentiating mesothelioma from other malignancies, but rather is useful when the cellular infiltrate has been confirmed as being mesothelial in origin in the first instance (8, 14, 27). Due to the greater expense and longer turnaround time of p16 FISH, an immunohistochemical alternative has been attempted. However, IHC loss of p16 is not specific for CDKN2A homozygous deletion and there is limited concordance between the loss of p16 IHC and homozygous deletion of CDKN2A (7, 32).

Homozygous deletions of p16 are considered to be relatively specific for MPM, however, point mutations and DNA methylation may occur in benign mesothelial cells. p16 is present in all cells and is involved in a normally functioning cellular cycle. Studies have reported a p16 deletion in approximately 80% of MPM cases, with 90–100% in sarcomatoid types, and approximately 70% of epithelioid and mixed type mesotheliomas (2, 33).

As touched on previously, one pitfall of using the presence of a p16 deletion in the diagnosis of MPM is that there exists the occasional case where p16 protein expression is maintained despite the presence of a p16 gene deletion, and vice versa. This is although multiple studies showing a statistically good association between p16 deletion and lack of p16 protein expression. Numerous factors could potentially explain this such as assay conditions, interpretation of results, and other variables. A homozygous deletion of p16 has strongly been associated with a shorter survival time in MPM patients (31).

The MTAP gene exists in close proximity to the p16 locus at 9p21. The protein produced by the MTAP gene is important for AMP and methionine salvage pathways. MTAP FISH has not been adopted into routine clinical practice yet. Using FISH analysis, both of these genes have been reported in many cases to be deleted together (8). MTAP has been reported by one study to have a loss rate of 23% in reactive mesothelial cases, indicating a lower than ideal specificity. The same study indicated MTAP may be useful only for separating benign from malignant mesothelial tumors (34). However, more recent studies have found much higher specificities and sensitivities. One study (7) which used a lower threshold for positive staining (60% of cells) found that MTAP loss had a specificity of 100% in addition to a reasonable sensitivity. This study reported a retention of MTAP in up to 32% of tumor cells in 21 of 23 of their confirmed mesothelioma cases. The other cases had loss rates greater than 80%.

Berg et al. (14) found a sensitivity of 90% and specificity of 100% for MPM when a combination of MTAP and BAP1 IHC was used to separate benign from malignant mesothelial proliferations. This study, as well as Chapel et al. (12), also found a good concordance (84%) between MTAP loss by IHC and p16 deletion by FISH. Churg et al. (5) and Kinoshita et al. (11) also found MTAP IHC could be used in cyotology cell-block preparations reliably. However, further research into the reproducibility of MTAP IHC is required to further validate its use into clinical practice.

Neurofibromatosis type 2 gene (NF2), located on chromosome 22q12, can also be analyzed by FISH. It has been found that hemizygous loss of NF2 is associated with invasiveness, proliferation of tumor tissue, and migration. NF2 has been shown to be a useful prognostic marker in mesothelioma of peritoneal origin in Singhi et al. (35), however, there was no deletion in cases of MPM seen in Berg et al. (14). Thurneysen et al. (36) found NF2 to be mutated in only 50% of pleural mesotheliomas. Therefore, although useful in theory, a loss of the NF2 gene by FISH is not advantageous in the diagnosis malignant pleural mesothelioma (MPM).

### Epigenetic Changes in MPM

One of the most emerging areas in the diagnosis of malignant mesothelioma is the use of epigenetic markers (39). It has long been suggested that epigenetic change play a pivotal role in the development of malignant pleural mesothelioma from chronic pleural inflammatory processes (40). Epigenetic modifications such as methylation, acetylation, ubiquination, phosphorylation, and SUMOylation, can either activate or repress gene expression (41). DNA methylation is considered to be an important epigenetic modification in the development of MPM (41). It involves the addition of a methyl group to the 5′ position of the cytosine usually next to a guanine base (CpG methylation). Methylation of CpG dinucleotide subsequently lead to the suppression of tumor suppressor genes that is thought to have a major role not only MPM, but many cancers (40, 42). Histone acetylation/deacetylation and methylation/demethylation are also well-researched areas of epigenetic medicine (43). These modifications are highly dynamic in response to environmental stimuli, and are also thought to play a major role in MPM in which a decrease in acetylation of certain histones has been reported (44). It has also been found that there is a significant upregulation of another entity, long-coding RNAs (lncRNAs), in mesothelioma. They are a more recently discovered class of RNAs of >200 nucleotides in length which act as decoys and scaffolds, and have been found to control every level of gene expression (39). Micro RNA (miRNAs) are another form of non-coding RNAs which have a post-transcriptional role in gene expression. Various oncogene miRNAs have been found to be upregulated, while at the same there is active and existing research into tumor suppressor miRNAs which have been downregulated, both associated with MPM (45, 46). Circular RNAs (circRNA) are considered to be the newest area within the realm of MPM epigenetic research. They act as miRNA sponges, thus also regulating gene expression, and in studies have been detected in human plasma samples, making future use in diagnosis feasible (47, 48).

These epigenetic markers discussed hold great promise with regard to MPM diagnosis and prognosis (49). Kristensen et al. (48) found a very large number of loci to be epigenetically altered in MPM and that the degree of asbestos exposure is proportional to the degree of epigenetic alteration. It is known that chronic inflammation and increased reactive oxygen species (ROS) production associated with lung asbestosis drives tumorigenesis. Tumor necrosis factor-alpha (TNF-α) and high-mobility group box 1 protein (HMBG-1), being released from macrophages and from the mesothelium respectively, are some of the drivers of the chronic inflammatory process (50) and induction of tumour suppressor gene (TSG) silencing (51). Many human malignancies are known to feature dysregulation of epigenetic control, and can have anomalous promoter methylation and histone modifications (52). It has been hypothesized that asbestos particles can become coated in iron-containing proteins, which is considered to be the main driver of inactivation of TSGs and the appearance of epigenetic alterations. An increased DNA methylation status has previously been found to be implicated with a shorter survival, and a greater number of methylated genes has been found in late-stage MPM and the sarcomatoid variant of MPM (38). Epigenetic markers are not currently utilized in the diagnosis of MPM. However, further research into the area (from a clinical perspective) is likely to, in the future, complement histological morphology, IHC, and FISH when diagnosing MPM in a clinical setting.

#### DNA Methylation

From an epigenetic point of view, DNA promoter methylation is considered to be a factor in many cancers, including MPM (49). DNA methylation is currently the most researched area of epigenetics, and recent research has highlighted examples of important tumour suppressor gene (TSG) methylation, or silencing, which contributes to the development of MPM (40, 52). Furthermore, such markers can be detected in patient plasma samples, which holds promise for future use in MPM diagnosis, treatment monitoring, and prognostication (40). Methylation is considered to be a major mechanism through which dynamic changes in gene expression are mediated during normal cellular differentiation and homeostasis, as well being involved in other functions such as repression of imprinted alleles, and expression of germ cell restricted genes, retroviral sequences, and repetitive DNA. CpG dinucleotides (CpG islands) exist in clusters in promoters in over half of all genes, and these generally exist in an unmethylated state. This state equates to a relaxed (euchromatic) structure which is transcriptionally active (38, 41). CpG islands can also exist elsewhere in the genome, which are typically hypermethylated in normal cells. In general during malignant transformation, epigenetic silencing (methylation) of certain genes occurs most commonly due to aberrant functioning of the elements of the DNA methylation machinery. Many of these genes are tumour suppressors (TSGs), which contributes to cancer transformation (40, 52).

Asbestos exposure is thought to trigger the methylation process in MPM via various mechanisms. Numerous studies of human cancers showing dysfunctional DNA have revealed high rates of aberrant promoter methylation in specific cancers (53). It has been found that the degree of DNA methylation is proportional to the degree of asbestos exposure, and links have been made between high amounts of methylation and increasing severity of disease (44). A family of proteins known as secreted frizzled-related proteins (SFRPs) are an example of proteins that have become epigenetically silenced, or methylated, in a range of cancers, including MPM (54). Methylation of various SFRP genes (SFRP1, SFRP2, SFRP4 and SFRP5) have been shown to contribute to MPM via gene silencing. These genes have tumor suppressive ability through their antagonism of the Wnt pathway. Wnt signaling is known to be upregulated in many cancers including colon and MPM, perhaps being activated by the chronic inflammatory processes associated with these diseases (40, 55). Cheng et al. (40) found that exposure of non-malignant cells to asbestos caused increased DNA methylation of the promoter of SFRP1/2 genes as well as a downregulation of mRNA expression of those genes. The same study also found SFRP2/5 genes may have tumor suppressor potential as their promoters tend to be hypermethylated in MPM which leads to downregulation. Also, it has been well-known that various biomarkers can be detected via non-invasive tests such as sputum, urine, plasma, and stool samples, with cancers including lung, bladder, breast, and colon being detected respectively (56–59). With regard to MPM, Cheng et al. (40) found that methylated SFRPs can indeed be detected in patient plasma samples, however, this finding was hindered by a relatively small sample size in this particular study. Therefore, further research into this area would be advantageous to further elucidate the validity of this result.

One recent study of a relatively large cohort of patients investigated DNA methylation markers in the peripheral blood as a tool in the diagnosis of MPM (60). When compared to the control group, the study found more than 800 differentially methylated CpG sites in the MPM group of patients. It identified three major hypomethylated CpGs (FOXK1, MYB, and TAF4), and four major hypermethylated CpGs (CXCR6/FYC01, TAP1, MORC2, and LIME1). It was found that these seven differentially methylated CpGs had diagnostic value, and univariate regression analysis showed these results were consistent across the different MPM histotypes (60).

ZIC1 is an example of a gene which is often silenced via DNA hypermethylation. This gene is involved in the process of apoptosis and encodes a group of zinc-finger transcription proteins. These proteins play a critical role in neural tube development and other embryological processes (61). Cheng et al. (42) found that ZIC1 was frequently absent in MPM cell lines, while present in normal mesothelial cell lines. The study used decitabine to demethylate samples or a plasmid expression construct to restore ZIC1 expression, and found that doing this had a similar role in the way of inhibition of the Wnt signaling pathway (62). This suggests ZIC1 functions as a TSG in malignant mesothelioma. ZIC1 has also been found to be silenced in gastrointestinal cancers such as gastric and colorectal cancers (42). Cheng et al. (42) found promoter methylation of ZIC1 in the majority of MPM tumor samples. This study also provides evidence that ZIC1 can control and target the expression of certain miRNAs. Some miRNAs, such as the miR-200 cluster, are downregulated which leads to increased Wnt signaling (63). Other miRNAs have been found to be upregulated in MPM, such as those of the miR-17-92 cluster, which are associated with increased cell proliferation and migration (22, 41). Cheng et al. (42) found that certain miRNAs namely miR23a and miR27a, which are normally negatively regulated by ZIC1, were found to be expressed at higher levels in MPM patients and were associated with a shorter survival time. miRNA alterations will be discussed in further detail a later section.

#### lncRNA Alterations

There is also evidence suggestive that aberrant expression of long non-coding RNAs (lncRNAs) have a significant influence on the development of cancers such as malignant mesothelioma (39). These more recently discovered group of non-coding RNAs are generally >200 nucleotides in length and play a role in gene transcription. They tend to have multiple roles including the control of post-transcriptional gene regulation, RNA maturation, regulation of chromatin structure, and regulation the activity of transcriptional factors (39). lncRNAs are important signaling molecules in that have the ability to respond to specific stimuli at specific times and therefore often act as markers for biologically important cellular events. It has also been suggested that lncRNAs act as decoys which have the ability to negatively regulated effector proteins, thus regulating transcription this way. The advent of next generation sequencing has provided useful information regarding lncRNAs and their important role in cancer development. lncRNAs tend to have high tissue specificity when used in the diagnosis of numerous other cancers. For example, the non-coding RNA, prostate cancer antigen 3 (PCA3), has been shown to have a higher sensitivity and specificity than PSA in the diagnosis of prostate cancer, and is even detectable in the urine of these patients. Other examples include the lncRNA highly upregulated in liver cancer (HULC) has been detected in hepatocellular carcinoma (HCC), and MALAT1 which has been shown to be prognostic in early stage lung adenocarcinoma. Although these markers are still in their research stages, they will likely have important diagnostic roles in the future (64).

Wright et al. (39) found there was consistent upregulation of lncRNAs in MPM, with significant potential to separate benign from malignant pleural disease. This study found that MPM lncRNA expression was dysregulated when compared to normal mesothelium, with lncRNAs known as AK130275, AK129685, EF177379, BX648695, NR003584 and AF268386 all being upregulated in the former entity when compared to the latter. The study also managed to establish a panel of lncRNAs with the ability to distinguish between reactive mesothelium and MPM with a relatively high sensitivity of 71.1% and a specificity of 100%. Markers were detectable in the tissue samples of patients, in FFPE as well as fresh frozen forms, indicating that lncRNAs potentially have future diagnostic and prognostic utility (39).

#### miRNA Alterations

Mi-RNAs are relatively small, non-coding segments of RNA which have an important post-transcriptional role in expression of genes. miRNAs are encoded by their own set of genes and they have the potential to affect many cellular processes such as apoptosis and cellular proliferation (65). miRNAs suppress translation and downregulate the targets of mRNA and thus have the ability to alter the levels of transcriptional and post-transcriptional mRNA (46). It has been found that miRNAs can function as tumor suppressors as well as oncogenes. miRNAs can also function as oncogenes by negatively regulating TSGs or genes that control cell differentiation or apoptosis (45).

Data has found that in patients with both mesothelioma and asbestosis, there is a downregulation of the expression of cell-free miRNAs circulating in the plasma, again indicating feasible use in the future in the diagnosis and prognostication of MPM. miRNAs also have been found to be very stable within the body and in stored samples making them attractive biomarkers for analysis (45, 66). Further studies with larger patient cohorts to further assess clinical relevance are needed as available data is still limited and results often vary. Various miRNA markers which have been covered in the literature are outlined below. Table 5 outlines some of the circulating miRNAs which have been covered in the literature whereas Table 6 outlines some of the miRNAs which have been studied on a FFPE or frozen tissue basis.

**TABLE 5:** Examples of circulating miRNAs in MPM.

	Expression in MPM	Sample size	Sensitivity and specificity	References
miR-16	Downregulated	MPM patients (*N* = 32), Controls with non-cancerous pulmonary disease (*N* = 15), Asbestos-exposed (*N* = 14), MPM – FFPE (*N* = 24)	86.7% sensitivity, 82.2% specificity	Mozzoni et al. (45)
miR-17	Downregulated	MPM patients (*N* = 32), Controls with non-cancerous pulmonary disease (*N* = 15), Asbestos-exposed (*N* = 14), MPM – FFPE (*N* = 24)	80.0% sensitivity, 84.4% specificity	Mozzoni et al. (45)
miR-126	Downregulated	MPM patients (*N* = 44), Healthy volunteers (*N* = 50), Asbestos-exposed (*N* = 196)	80% sensitivity, 97.8% specificity (45); 73% sensitivity, 74% specificity (74)	Mozzoni et al. (45), Santarelli et al. (74)
miR-486	Downregulated	MPM patients (*N* = 32), Controls with non-cancerous pulmonary disease (*N* = 15), Asbestos-exposed (*N* = 14), MPM – FFPE (*N* = 24)	80.0% sensitivity, 89.1% specificity	Mozzoni et al. (45)

**TABLE 6:** Examples of miRNAs in MPM – from studies involving FFPE/frozen tissue samples.

	Expression in MPM	Ability of each microRNA to individually differentiate MPM	Comments *adenocarcinoma and other benign pleural disease combined
miR-143	Downregulated (75)	AUC 0.66 (95% CI: 0.50, 0.82) MPM from other pleural diseases* OR −0.30 (95% CI −0.62, 0.01) (75)	Good discriminator of MPM from other pleural disease* (75)
miR185-5p	Downregulated (76)	Log_2_(FC) −2.742, *P*-value 2.22 × 10^–4^	De Santi et al. (76) found statistically significant down-regulation in MPM samples when compared to normal pleural tissue controls.
miR197-3p	Downregulated (76)	Log_2_(FC) -2.540, *P*-value 3.72 × 10^–7^	De Santi et al. (76) found statistically significant down-regulation in MPM samples when compared to normal pleural tissue controls
miR-200c	Downregulated (75)	AUC of 0.79 (95% CI: 0.66, 0.92) MPM from other pleural diseases* OR: -0.87 (95% CI: −1.49, −0.24) (75)	Birnie et al. (75) found miR-200c to be the best discriminator of MPM from other pleural disease*
miR-299-5p	Downregulated (76)	Log_2_(FC) −1.239, P-value 0.0014	De Santi et al. (76) found statistically significant down-regulation in MPM samples when compared to normal pleural tissue controls
miR-139-5p	Upregulated (75)	AUC 0.65 (95% CI: 0.50, 0.81) MPM from other pleural diseases* OR: 0.42 (−0.01, 0.85) (75)	Good discriminator of MPM from other pleural disease* (75)
miR-210	Upregulated (75)	AUC 0.72 (95% CI: 0.58, 0.87) MPM from other pleural diseases* OR: 0.59 (95% CI: 0.07, 1.11) (75)	Good discriminator of MPM from other pleural disease* (75) Birnie et al. (75) showed that a combination of miR-200c, miR-210, and miR-143 resulted in an AUC of 0.92 (95% CI: 0.84, 0.99) – providing evidence that the combination is better at differentiating MPM from other pleural disease compared any of the mentioned microRNAs alone.

##### miRNA-16

miRNA-16 is considered to be a major tumor suppressor gene. It is located at chromosome 13q14 and has been shown to be involved in the cell cycle. It is implicated in various cancers such as gastric, breast, and lung cancer, and has been found to act as a tumor suppressor via suppressive effects on the cell cycle (67–69). miRNA-16 has recently emerged as a G1 cell cycle checkpoint, and it has been implicated in the regulation of cyclin D1 and E proteins through their 3′UTR. Reid et al. suggested that there was a consistent downregulation of the miRNA-15/16 family in all MPM tumors, when compared with normal mesothelial tissue samples (64). It also found that restoring normal levels of miRNA-16 *in vitro* had an inhibitory effect on malignant mesothelial cell types (45, 64). These studies highlight the potential usefulness of miRNA-16 as both a diagnostic, treatment, and prognostic marker for MPM.

##### miRNA-17

One study found that miRNA-17 is downregulated in MPM patients when compared to controls, supporting the theory that miRNA-17 has tumor suppressor function (45). It has been found in other studies to have both tumor suppressor and oncogenic function, depending on the cellular context. miRNA-17 is also downregulated in other fibrotic diseases such as idiopathic pulmonary fibrosis and hepatic fibrosis, which supports the concept that this gene is implicated in the control of the fibrotic component of MPM.

##### miRNA-126

miRNA-126 is downregulated in many cancer types such as pancreatic, lung, and kidney cancers (45, 70, 71). It appears to be the most frequently studied miRNA in the literature. It is generally considered to be a tumor suppressor, with some oncogenic roles also identified. miRNA-126 has been implicated in the process of angiogenesis, with downregulation of this protein leading to increased production of VEGF, which in turn promotes vascularization (72). Increased expression of VEGF and its markers is correlated with the degree of vascularization in tumorigenesis and is implicated in increased metastatic risk. Mozzoni et al. (45) demonstrated a downregulation of miRNA-126 in the plasma samples of MPM patients, as well as this inverse relationship with VEGF expression, implying amplified tumor growth and higher risk of metastasis.

##### mi-RNA-486

mi-RNA-486 is strongly downregulated in patients with asbestosis and MPM. It has been demonstrated that mi-RNA486 has strong antifibrotic activity and decreased expression is associated with fibrotic lung diseases. Since the development of mesothelioma is generally associated with the development of a fibrotic process, it can be expected that miRNA-486 could be used as a novel marker in asbestosis diagnosis or as a treatment of MPM (73).

MPM is still considered to be extraordinarily resistant to therapy, and further research into the realm of epigenetic markers would potentially yield highly useful diagnostic tests and treatments for this disease.

#### Histone Modifications

Much of the current research into histone modifications in MPM is within the realm of treatment, rather than diagnostics. Histones are a group of basic proteins that provide structure and regulatory function to chromatin. The role of chromatin is to pack DNA efficiently within the nucleus inside each cell, and comprises DNA, RNA, and both histone and non-histone elements. Proteins called nucleosomes make up chromatin which comprise 147 base pairs of DNA and are “wound” around histone subunits (77). A process called histone acetylation increases the amount of negative charge via the addition of an acetyl group. This leads to relaxation of chromatin via repulsion of DNA, and subsequent activation of gene expression. Studies have shown that chromatin alteration resulting from histone acetylation is an important factor in the neoplastic process. Histone deacetylation of tumor suppressors is considered a major cause of tumorigenesis. Acetylation is mediated by a range of histone acetyltransferases (HATs) whereas histone decacetylation is considered to be mediated by four main classes of histone deacetylases (HDACs). There are also non-histone protein targets of HATs and HDACs such as Hsp90, p53, SP1, and HDAC1 (78). Histone deacetylation causing and thus transcriptional silencing and inactivation of tumor suppressors is considered a major cause of cancer growth. For example, the retinoblastoma gene (Rb) is one such TSG which has been found to be suppressed by deacetylated histones at promoter regions, leading to cancer growth. Specifically in MPM, a decrease of acetylation of histones H3 and H4 has been reported (79). HDAC inhibitor therapy in the literature has shown an increase in acetylation of H3 and H4 histones, which may show promise as a future treatment for MPM (77, 79, 80).

As alluded to earlier, research in this area has centered mostly on emerging treatment modalities for MPM, namely HDAC inhibition therapy. *In vitro* biologic effects of HDAC inhibitors have included cell cycle arrest, angiogenic inhibition, immunomodulation, and direct acetylation of signaling intermediates and transcription factors (81). Shao et al. (82) first brought to light the significance of histone acetylation in MPM through research into the regulation of tumor suppressor Wilms tumor-1 (WT-1). The study found overexpression of HDAC4 and HDAC5 and a decrease in WT-1 activity and associated mRNA expression in 293T cells. This decrease was reversed by cotransfection of the HAT P300, which also increased H3 acetylation at the WT-1 intronic enhancer.

#### Circular RNA

Circular RNAs (circRNAs) are another member of the non-coding genome and were first discovered just over 40 years ago. These differ from linear mRNA, being that they are covalently closed single-stranded RNAs lacking 5′ caps or 3′ poly(A) tails, which are generated from pre-mRNAs by a process known as backsplicing (83, 84). Research into this area is still in its infancy and its role in tumorigenesis is only just starting to be understood. Because circRNAs in general have other functions independent of their host genes, and there is as of yet no agreed standard of researching and naming circRNAs, research into the area has been challenging (48). circRNA research is one of the most emerging areas in epigenetics, especially within the realm of mesothelioma diagnosis. This particular type of RNA is considered to be endogenous RNA involved in normal cellular differentiation and tissue homeostasis. circRNA molecules are stable entities and have gene-regulatory function through their action as miRNA “sponges” (48). To date, there have been numerous circRNAs identified in the literature which have been shown to be dysregulated in numerous human cancers. Malignancies of the liver, lung (including MPM), breast, prostate, bladder, colorectal, ovarian, central nervous system, and stomach, as well as several hematological malignancies have been shown to have aberrancy of expression of various circRNA markers (85). circRNA markers can be detected in human blood and saliva samples, highlighting their potential to be exploited as biomarker candidates for the development of novel non-invasive cancer diagnostic techniques. Furthermore, circRNAs are desirable candidates for use as blood-based biomarkers as they have a longer associated half-life and are resistant to exonuclease-mediated degradation compared to their linear counterparts (86–88).

There are many proposed mechanisms relating to the effect of circular RNA on tumorigenesis. Firstly, as previously alluded to, they are likely to function as miRNA “sponges” (decoys). When miRNAs bind to circRNAs, target mRNAs may be released from degraded miRNA which may result in more efficient translation (89). Normally miRNAs primarily bind to the 3′ untranslated regions (UTR’s) of specific mRNA targets, whereby they function as post-transcriptional regulators of gene expression for cellular events such as cell proliferation, migration, differentiation, and apoptosis (90, 91). In the recent circRNA profiling study, a majority of circRNAs are now known to harbor complementary binding sites of “tumor suppressor” miRNAs, implying that they are capable of binding to and inactivating the miRNA; thus impeding their interaction with their mRNA targets (92). This is supported by growing evidence that have identified a clear trend in over- and under-expression of circRNAs and miRNAs, respectively. in many cancer types, including lung adenocarcinoma, colorectal, gastric, breast cancer (93) and mesothelioma. Additionally, circRNAs harbor binding sites for various enzymes, with their substrates acting as scaffolds between two or more proteins (94). Most circRNA exists in the cytosol of cells, with some being retained within the nucleus, which brings to light a third mechanism in tumorigenesis. Here in the nucleus circRNA can interfere with transcription or foster alternate splicing. These exon-intron circRNAs have been shown to have a relationship to RNA polymerase II thus promoting, via interaction with U1 snRNP, the transcription of associated genes (95). This latter mechanism is still in very early research stages and it is still not entirely known whether intron-exon circRNAs become dysregulated in cancer (96).

The involvement of aberrant circRNA expression in the development of lung cancer has been a relatively well and recently researched area. A well-characterized circRNA in this form of cancer, named circITCH, is considered to negatively affect cellular proliferation and inhibit components of the cell cycle. Inhibition of the Wnt/beta-Catenin pathway is an example of one of these mechanisms. Wan et al. (47) performed a study of 78 patients and found circITCH to be downregulated in lung cancer tissues. Another example is the involvement of a circRNA named ciRS-7 (also known as CDR1as) in colorectal cancer. It is among one of the first and also one of the most studied circRNAs (47). It is present in many human tissues and directly targets many oncogenes, and is thus involved in many human cancers (89). It has been found that in colorectal cancer, upregulation of ciRS-7 is present, which is in contrast to most circRNAs which are downregulated (89, 97).

The involvement of aberrant circular RNA expression in malignant pleural mesothelioma (MPM) is a scarcely researched area in medicine. One study (98) found several circRNAs with associated tumor-suppressor microRNA targets to be upregulated in MPM. It involved the isolation of RNA from a series of 9 MPM patients, as well as 2 patients with normal mesothelium. In our preliminary study, we found upregulation of 290 circRNAs in the MPM samples, with the functionally relevant ones being PHKB, SLC45A4, ARHGEF28, FBXW4, TAF15, PLEKHM1, RALGPS1, STIL, L3MBTL4, ANKRD27, NHS, ILKAP, and PTK2. These circRNAs harbored predicted tumor suppressor binding sites for miRNAs miR-16, miR-15a, miR-15b, miR-34a, miR-34b, miR-34c and miR-137 (manuscript in preparation). Further research is necessary to better detection method within patient plasma samples which could establish clinical relevance in the realm of MPM with regard to diagnosis, prognosis, and treatment.

Circular RNA research in the realm of cancer diagnosis has been rather limited relative to other areas of epigenetics, mostly due to practical matters that hamper their detection by standard molecular biology techniques. For example, when conventional reverse transcriptase-quantitative PCR assays are used, a linear RNA genome is generally used as a template for primer design; these PCR assays unfortunately do not distinguish between circular and linear RNA (48). Also, specific circRNAs need to be actively searched for in order to be identified. Another problem is that most protocols for RNA sequencing need to remove ribosomal RNA from the sample via a poly(A) purification step. circRNAs lack a poly(A) tail, making refinement of the sample more complicated.

Due to growing interest in circRNA cancer biology and the advent of innovative bioinformatic technology, there are a range of emerging web-based circRNA databases that provide a comprehensive characterization of more than one hundred thousand circRNAs (99); including detailed sequence profiles, their different isoforms, predicted miRNA binding sites, and associated diseases. The circRNA database, CircInteractome, not only provides detailed circRNA information, but is currently the only database that provides an innovative web-based tool for designing highly specific divergent primers for the detection and quantification of circRNA candidates (100, 101). The divergent primers are able to be uniquely designed to span and target the known sequence of the circRNA backsplice exonic junction region, which upon their application to quantitative PCR, specifically amplify the circRNAs and not the counterpart linear RNA. Hence, in contrast to conventional quantitative PCR methods, divergent primer-based PCR is capable of distinguishing between circRNA and linear RNA. This has therefore led to the emergence of novel divergent primer-based quantitative PCR techniques that have proven to be effective at detecting, validating and quantitating circRNAs in a range of recent studies (102–104). As mentioned previously, circRNA expression in MPM remains a relatively uncharted area of research, and given the growing evidence indicating an upregulation of circRNA candidates in MPM, the novel circRNA PCR technique represents a useful means for the detection and quantification of the upregulated circRNA targets in MPM patient biospecimens. More specifically, such a technique is desirable for the detection and quantification of circRNA candidates in MPM patient blood samples for their potential development into non-invasive blood-based biomarkers, which in the case of MPM, would constitute a significant advance compared to the current limitations of invasive biopsy-based diagnostic techniques.

## Conclusion

Malignant pleural mesothelioma (MPM) is an aggressive tumor which arises from the mesothelial cells lining the pleural cavity. It is associated with a relatively poor life expectancy and definitive diagnosis is still considered to be difficult to attain. Diagnosis of MPM based on histopathological features can be difficult as it often can resemble other tumors, and typical indicators of malignancy such as cellular atypia and mitotic bodies are generally not useful. Immunohistochemical markers are essential to the diagnosis, with an initial mesothelial cell panel which may include calretinin, CK 5/6, WT-1 and D2-40 being used to differentiate mesothelial cell origin from metastatic carcinomas. The latter generally stain positive for the markers such as CEA monoclonal, BerEP4, MOC-31, Claudin-4, and B72.3. Another important step in MPM diagnosis is differentiating it from reactive mesothelial proliferation. BAP1 IHC and MTAP IHC now have sufficient evidence behind their use in diagnosis and are being widely utilized in clinical practice today to differentiate between reactive mesothelial proliferation and MPM. While individually their sensitivity for MPM has shown mixed results, both have excellent specificity. When used together their sensitivity rises considerably and a similar effect is observed when each is used in conjunction with FISH testing for p16 loss. The sensitivity rises above 90% for MPM over reactive mesothelial cells. FISH testing for p16 loss is another diagnostic modality which has successfully been incorporated into clinical practice. Research into the use of epigenetic markers in the diagnosis of MPM has shown very promising results in the literature. DNA methylation markers are currently the most researched area in MPM epigenetic diagnosis. Certain hyper- and hypomethylated CpG sites have been found to carry diagnostic value in MPM, with markers being detected in patient plasma. Other epigenetic markers such as certain long non-coding RNAs, micro RNAs, histone acetylation markers, and circular RNA markers similarly have shown promise in MPM diagnosis. Many studies currently exist strongly supporting the clinical relevance of incorporating epigenetic markers into the diagnosis of other cancers, however, in the case of MPM this evidence is still in its early stages. Although evidence so far is encouraging, larger studies with larger cohorts of patients are needed to assess the use of these epigenetic markers in MPM diagnosis to clarify clinical relevance.

## Author Contributions

ER drafted the manuscript. KL and YC conceived and edited the manuscript. BJ edited the manuscript. All authors contributed to the article and approved the submitted version.

## Conflict of Interest

The authors declare that the research was conducted in the absence of any commercial or financial relationships that could be construed as a potential conflict of interest.

## References

[1] Selikoff IJ Churg J Hammond EC. Asbestos exposure and neoplasia. *JAMA.* (1964) 188:22–6. 14107207 10.1001/jama.1964.03060270028006

[2] Husain AN Colby TV Ordonez NG Allen TC Attanoos RL Beasley MB Guidelines for pathologic diagnosis of malignant mesothelioma 2017 update of the consensus statement from the international mesothelioma interest group. *Arch Pathol Lab Med.* (2018) 142:89–108. 10.5858/arpa.2017-0124-ra 28686500

[3] Baas P Fennell D Kerr KM Van Schil PE Haas RL Peters S Malignant pleural mesothelioma: ESMO clinical practice guidelines for diagnosis, treatment and follow-up. *Ann Oncol.* (2015) 26 (Suppl. 5):v31–9. 10.1093/annonc/mdv199 26223247

[4] Beebe-Dimmer JL Fryzek JP Yee CL Dalvi TB Garabrant DH Schwartz AG Mesothelioma in the United States: a surveillance, epidemiology, and end results (SEER)-Medicare investigation of treatment patterns and overall survival. *Clin Epidemiol.* (2016) 8:743–50. 10.2147/clep.s105396 27822122 PMC5087771

[5] Churg A Nabeshima K Ali G Bruno R Fernandez-Cuesta L Galateau-Salle F. Highlights of the 14th international mesothelioma interest group meeting: Pathologic separation of benign from malignant mesothelial proliferations and histologic/molecular analysis of malignant mesothelioma subtypes. *Lung Cancer.* (2018) 124:95–101. 10.1016/j.lungcan.2018.07.041 30268487

[6] Nasu M Emi M Pastorino S Tanji M Powers A Luk H High incidence of somatic BAP1 alterations in sporadic malignant mesothelioma. *J Thorac Oncol.* (2015) 10:565–76. 10.1097/jto.0000000000000471 25658628 PMC4408084

[7] Hida T Hamasaki M Matsumoto S Sato A Tsujimura T Kawahara K Immunohistochemical detection of MTAP and BAP1 protein loss for mesothelioma diagnosis: comparison with 9p21 FISH and BAP1 immunohistochemistry. *Lung Cancer.* (2017) 104:98–105. 10.1016/j.lungcan.2016.12.017 28213009

[8] Illei PB Rusch VW Zakowski MF Ladanyi M. Homozygous deletion of CDKN2A and codeletion of the methylthioadenosine phosphorylase gene in the majority of pleural mesotheliomas. *Clin Cancer Res.* (2003) 9:2108–13. 12796375

[9] Savic S Franco N Grilli B Barascud Ade V Herzog M Bode B Fluorescence in situ hybridization in the definitive diagnosis of malignant mesothelioma in effusion cytology. *Chest.* (2010) 138:137–44. 10.1378/chest.09-1951 20139227

[10] Chiosea S Krasinskas A Cagle PT Mitchell KA Zander DS Dacic S. Diagnostic importance of 9p21 homozygous deletion in malignant mesotheliomas. *Mod Pathol.* (2008) 21:742–7. 10.1038/modpathol.2008.45 18327208

[11] Kinoshita Y Hida T Hamasaki M Matsumoto S Sato A Tsujimura T A combination of MTAP and BAP1 immunohistochemistry in pleural effusion cytology for the diagnosis of mesothelioma. *Cancer Cytopathol.* (2018) 126:54–63. 10.1002/cncy.21928 29053210

[12] Chapel DB Schulte JJ Berg K Churg A Dacic S Fitzpatrick C MTAP immunohistochemistry is an accurate and reproducible surrogate for CDKN2A fluorescence in situ hybridization in diagnosis of malignant pleural mesothelioma. *Mod Pathol.* (2020) 33:245–54. 10.1038/s41379-019-0310-0 31231127

[13] Hida T Hamasaki M Matsumoto S Sato A Tsujimura T Kawahara K BAP1 immunohistochemistry and p16 FISH results in combination provide higher confidence in malignant pleural mesothelioma diagnosis: ROC analysis of the two tests. *Pathol Int.* (2016) 66:563–70. 10.1111/pin.12453 27614970

[14] Berg KB Dacic S Miller C Cheung S Churg A. Utility of methylthioadenosine phosphorylase compared with BAP1 immunohistochemistry, and CDKN2A and NF2 fluorescence in situ hybridization in separating reactive mesothelial proliferations from epithelioid malignant mesotheliomas. *Arch Pathol Lab Med.* (2018) 142:1549–53. 10.5858/arpa.2018-0273-oa 30059257

[15] Henderson DW Reid G Kao SC van Zandwijk N Klebe S. Challenges and controversies in the diagnosis of mesothelioma: Part 1. Cytology-only diagnosis, biopsies, immunohistochemistry, discrimination between mesothelioma and reactive mesothelial hyperplasia, and biomarkers. *J Clin Pathol.* (2013) 66:847–53. 10.1136/jclinpath-2012-201303 23814259

[16] Hjerpe A Ascoli V Bedrossian C Boon M Creaney J Davidson B Guidelines for cytopathologic diagnosis of epithelioid and mixed type malignant mesothelioma. Complementary statement from the International Mesothelioma Interest Group, also endorsed by the International Academy of Cytology and the Papanicolaou Society of Cytopathology. *Cytojournal.* (2015) 12:26. 10.4103/1742-6413.170726 26681974 PMC4678521

[17] Smith M Colby T. The diagnosis of thoracic malignant mesothelioma: practical considerations and recent developments. *Turk Patoloji Derg.* (2014) 30:1–10. 10.5146/tjpath.2014.01158 24448702

[18] Ordonez NG. Value of PAX8, PAX2, claudin-4, and h-caldesmon immunostaining in distinguishing peritoneal epithelioid mesotheliomas from serous carcinomas. *Mod Pathol.* (2013) 26:553–62. 10.1038/modpathol.2012.200 23196794

[19] Bishop JA Sharma R Illei PB. Napsin A and thyroid transcription factor-1 expression in carcinomas of the lung, breast, pancreas, colon, kidney, thyroid, and malignant mesothelioma. *Hum Pathol.* (2010) 41:20–5. 10.1016/j.humpath.2009.06.014 19740516

[20] Ordonez NG Sahin AA. Diagnostic utility of immunohistochemistry in distinguishing between epithelioid pleural mesotheliomas and breast carcinomas: a comparative study. *Hum Pathol.* (2014) 45:1529–40. 10.1016/j.humpath.2014.03.006 24816068

[21] Tacha D Zhou D Cheng L. Expression of PAX8 in normal and neoplastic tissues: a comprehensive immunohistochemical study. *Appl Immunohistochem Mol Morphol.* (2011) 19:293–9. 10.1097/pai.0b013e3182025f66 21285870

[22] Chapel DB Husain AN Krausz T McGregor SM. PAX8 expression in a subset of malignant peritoneal mesotheliomas and benign mesothelium has diagnostic implications in the differential diagnosis of ovarian serous carcinoma. *Am J Surg Pathol.* (2017) 41:1675–82. 10.1097/pas.0000000000000935 28877056

[23] Hjerpe A Abd-Own S Dobra K. Cytopathologic diagnosis of epithelioid and mixed-type malignant mesothelioma: ten years of clinical experience in relation to international guidelines. *Arch Pathol Lab Med.* (2018) 142:893–901. 10.5858/arpa.2018-0020-ra 30040460

[24] Churg A Sheffield BS Galateau-Salle F. New markers for separating benign from malignant mesothelial proliferations: are we there yet? *Arch Pathol Lab Med.* (2016) 140:318–21. 10.5858/arpa.2015-0240-sa 26288396

[25] Bott M Brevet M Taylor BS Shimizu S Ito T Wang L The nuclear deubiquitinase BAP1 is commonly inactivated by somatic mutations and 3p21.1 losses in malignant pleural mesothelioma. *Nat Genet.* (2011) 43:668–72. 10.1038/ng.855 21642991 PMC4643098

[26] Carbone M Ferris LK Baumann F Napolitano A Lum CA Flores EG BAP1 cancer syndrome: malignant mesothelioma, uveal and cutaneous melanoma, and MBAITs. *J Transl Med.* (2012) 10:179. 10.1186/1479-5876-10-179 PMC3493315 22935333

[27] Hwang HC Sheffield BS Rodriguez S Thompson K Tse CH Gown AM Utility of BAP1 immunohistochemistry and p16 (CDKN2A) FISH in the diagnosis of malignant mesothelioma in effusion cytology specimens. *Am J Surg Pathol.* (2016) 40:120–6. 10.1097/pas.0000000000000529 26448191

[28] Hwang HC Pyott S Rodriguez S Cindric A Carr A Michelsen C BAP1 Immunohistochemistry and p16 FISH in the Diagnosis of sarcomatous and desmoplastic mesotheliomas. *Am J Surg Pathol.* (2016) 40:714–8. 10.1097/pas.0000000000000616 26900815

[29] Sheffield BS Hwang HC Lee AF Thompson K Rodriguez S Tse CH BAP1 immunohistochemistry and p16 FISH to separate benign from malignant mesothelial proliferations. *Am J Surg Pathol.* (2015) 39:977–82. 10.1097/pas.0000000000000394 25634745

[30] Lo Iacono M Monica V Righi L Grosso F Libener R Vatrano S Targeted next-generation sequencing of cancer genes in advanced stage malignant pleural mesothelioma: a retrospective study. *J Thorac Oncol.* (2015) 10:492–9. 10.1097/jto.0000000000000436 25514803

[31] Lopez-Rios F Chuai S Flores R Shimizu S Ohno T Wakahara K Global gene expression profiling of pleural mesotheliomas: overexpression of aurora kinases and P16/CDKN2A deletion as prognostic factors and critical evaluation of microarray-based prognostic prediction. *Cancer Res.* (2006) 66:2970–9. 10.1158/0008-5472.can-05-3907 16540645

[32] Krasinskas AM Bartlett DL Cieply K Dacic S. CDKN2A and MTAP deletions in peritoneal mesotheliomas are correlated with loss of p16 protein expression and poor survival. *Mod Pathol.* (2010) 23:531–8. 10.1038/modpathol.2009.186 20081810

[33] Wu D Hiroshima K Matsumoto S Nabeshima K Yusa T Ozaki D Diagnostic usefulness of p16/CDKN2A FISH in distinguishing between sarcomatoid mesothelioma and fibrous pleuritis. *Am J Clin Pathol.* (2013) 139:39–46. 10.1309/ajcpt94jvwihbkrd 23270897

[34] Zimling ZG Jorgensen A Santoni-Rugiu E. The diagnostic value of immunohistochemically detected methylthioadenosine phosphorylase deficiency in malignant pleural mesotheliomas. *Histopathology.* (2012) 60:E96–105. 22394205 10.1111/j.1365-2559.2012.04196.x

[35] Singhi AD Krasinskas AM Choudry HA Bartlett DL Pingpank JF Zeh HJ The prognostic significance of BAP1, NF2, and CDKN2A in malignant peritoneal mesothelioma. *Mod Pathol.* (2016) 29:14–24. 10.1038/modpathol.2015.121 26493618

[36] Thurneysen C Opitz I Kurtz S Weder W Stahel RA Felley-Bosco E. Functional inactivation of NF2/merlin in human mesothelioma. *Lung Cancer.* (2009) 64:140–7. 10.1016/j.lungcan.2008.08.014 18835652

[37] Liu J Liao X Gu Y Fu L Zhao J Li L Role of p16 deletion and BAP1 loss in the diagnosis of malignant mesothelioma. *J Thorac Dis.* (2018) 10:5522–30. 10.21037/jtd.2018.08.59 30416802 PMC6196170

[38] Goto Y Shinjo K Kondo Y Shen L Toyota M Suzuki H Epigenetic profiles distinguish malignant pleural mesothelioma from lung adenocarcinoma. *Cancer Res.* (2009) 69:9073–82. 10.1158/0008-5472.can-09-1595 19887624

[39] Wright CM Kirschner MB Cheng YY O’Byrne KJ Gray SG Schelch K Long non coding RNAs (lncRNAs) are dysregulated in malignant pleural mesothelioma (MPM). *PLoS One.* (2013) 8:e70940. 10.1371/journal.pone.0070940 23976967 PMC3747266

[40] Cheng YY Mok E Tan S Leygo C McLaughlin C George AM SFRP tumour suppressor genes are potential plasma-based epigenetic biomarkers for malignant pleural mesothelioma. *Dis Markers.* (2017) 2017:2536187. 10.1155/2017/2536187 PMC5745727 29386699

[41] Dawson MA Kouzarides T. Cancer epigenetics: from mechanism to therapy. *Cell.* (2012) 150:12–27. 10.1016/j.cell.2012.06.013 22770212

[42] Cheng YY Kirschner MB Cheng NC Gattani S Klebe S Edelman JJ ZIC1 is silenced and has tumor suppressor function in malignant pleural mesothelioma. *J Thorac Oncol.* (2013) 8:1317–28. 10.1097/jto.0b013e3182a0840a 24457242

[43] Chi P Allis CD Wang GG. Covalent histone modifications–miswritten, misinterpreted and mis-erased in human cancers. *Nat Rev Cancer.* (2010) 10:457–69. 10.1038/nrc2876 20574448 PMC3262678

[44] Christensen BC Houseman EA Godleski JJ Marsit CJ Longacker JL Roelofs CR Epigenetic profiles distinguish pleural mesothelioma from normal pleura and predict lung asbestos burden and clinical outcome. *Cancer Res.* (2009) 69:227–34. 10.1158/0008-5472.can-08-2586 19118007 PMC2744125

[45] Mozzoni P Ampollini L Goldoni M Alinovi R Tiseo M Gnetti L MicroRNA Expression in malignant pleural mesothelioma and asbestosis: a pilot study. *Dis Markers.* (2017) 2017:9645940. 10.1155/2017/9645940 PMC5512053 28757678

[46] Treiber T Treiber N Meister G. Regulation of microRNA biogenesis and function. *Thromb Haemost.* (2012) 107:605–10. 10.1160/th11-12-0836 22318703

[47] Wan L Zhang L Fan K Cheng ZX Sun QC Wang JJ. Circular RNA-ITCH suppresses lung cancer proliferation via inhibiting the wnt/beta-catenin pathway. *Biomed Res Int.* (2016) 2016:1579490. 10.1155/2016/1579490 PMC5013215 27642589

[48] Kristensen LS Hansen TB Veno MT Kjems J. Circular RNAs in cancer: opportunities and challenges in the field. *Oncogene.* (2018) 37:555–65. 10.1038/onc.2017.361 28991235 PMC5799710

[49] Hussain SP Harris CC. Inflammation and cancer: an ancient link with novel potentials. *Int J Cancer.* (2007) 121:2373–80. 10.1002/ijc.23173 17893866

[50] Bani-Hani KE Gharaibeh KA. Malignant peritoneal mesothelioma. *J Surg Oncol.* (2005) 91:17–25. 15999348 10.1002/jso.20266

[51] Sun HH Vaynblat A Pass HI. Diagnosis and prognosis-review of biomarkers for mesothelioma. *Ann Transl Med.* (2017) 5:244. 10.21037/atm.2017.06.60 28706912 PMC5497115

[52] Jones PA Baylin SB. The fundamental role of epigenetic events in cancer. *Nat Rev Genet.* (2002) 3:415–28. 10.1038/nrg816 12042769

[53] Toyota M Issa JP. CpG island methylator phenotypes in aging and cancer. *Semin Cancer Biol.* (1999) 9:349–57. 10.1006/scbi.1999.0135 10547343

[54] Shi Y He B You L Jablons DM. Roles of secreted frizzled-related proteins in cancer. *Acta Pharmacol Sin.* (2007) 28:1499–504. 10.1111/j.1745-7254.2007.00692.x 17723183

[55] Lee AY He B You L Dadfarmay S Xu Z Mazieres J Expression of the secreted frizzled-related protein gene family is downregulated in human mesothelioma. *Oncogene.* (2004) 23:6672–6. 10.1038/sj.onc.1207881 15221014

[56] Hubers AJ Prinsen CF Sozzi G Witte BI Thunnissen E. Molecular sputum analysis for the diagnosis of lung cancer. *Br J Cancer.* (2013) 109:530–7. 10.1038/bjc.2013.393 23868001 PMC3738145

[57] Ward DG Baxter L Gordon NS Ott S Savage RS Beggs AD Multiplex PCR and next generation sequencing for the non-invasive detection of bladder cancer. *PLoS One.* (2016) 11:e0149756. 10.1371/journal.pone.0149756 26901314 PMC4762704

[58] Henderson MC Silver M Tran Q Letsios EE Mulpuri R Reese DE A noninvasive blood-based combinatorial proteomic biomarker assay to detect breast cancer in women over age 50 with BI-RADS 3, 4, or 5 assessment. *Clin Cancer Res.* (2019) 25:142–9. 30185421 10.1158/1078-0432.CCR-18-0843

[59] Russo G Patrignani A Poveda L Hoehn F Scholtka B Schlapbach R Highly sensitive, non-invasive detection of colorectal cancer mutations using single molecule, third generation sequencing. *Appl Transl Genom.* (2015) 7:32–9. 10.1016/j.atg.2015.08.006 27054083 PMC4803778

[60] Guarrera S Viberti C Cugliari G Allione A Casalone E Betti M Peripheral Blood DNA methylation as potential biomarker of malignant pleural mesothelioma in asbestos-exposed subjects. *J Thorac Oncol.* (2019) 14:527–39. 10.1016/j.jtho.2018.10.163 30408567

[61] Aruga J. The role of Zic genes in neural development. *Mol Cell Neurosci.* (2004) 26:205–21. 10.1016/j.mcn.2004.01.004 15207846

[62] Kohno H Amatya VJ Takeshima Y Kushitani K Hattori N Kohno N Aberrant promoter methylation of WIF-1 and SFRP1, 2, 4 genes in mesothelioma. *Oncol Rep.* (2010) 24:423–31. 10.3892/or_00000875 20596629

[63] Gee GV Koestler DC Christensen BC Sugarbaker DJ Ugolini D Ivaldi GP Downregulated microRNAs in the differential diagnosis of malignant pleural mesothelioma. *Int J Cancer.* (2010) 127:2859–69. 10.1002/ijc.25285 21351265 PMC2911512

[64] Reid G Pel ME Kirschner MB Cheng YY Mugridge N Weiss J Restoring expression of miR-16: a novel approach to therapy for malignant pleural mesothelioma. *Ann Oncol.* (2013) 24:3128–35. 10.1093/annonc/mdt412 24148817

[65] Lo Russo G Tessari A Capece M Galli G de Braud F Garassino MC MicroRNAs for the diagnosis and management of malignant pleural mesothelioma: a literature review. *Front Oncol.* (2018) 8:650. 10.3389/fonc.2018.00650 30622932 PMC6308141

[66] Sozzi G Pastorino U Croce CM. MicroRNAs and lung cancer: from markers to targets. *Cell Cycle.* (2011) 10:2045–6. 10.4161/cc.10.13.15712 21623159

[67] Bandi N Zbinden S Gugger M Arnold M Kocher V Hasan L miR-15a and miR-16 are implicated in cell cycle regulation in a Rb-dependent manner and are frequently deleted or down-regulated in non-small cell lung cancer. *Cancer Res.* (2009) 69:5553–9. 10.1158/0008-5472.CAN-08-4277 19549910

[68] Lages E Ipas H Guttin A Nesr H Berger F Issartel JP. MicroRNAs: molecular features and role in cancer. *Front Biosci (Landmark Ed).* (2012) 17:2508–40. 10.2741/4068 22652795 PMC3815439

[69] Wu WL Wang WY Yao WQ Li GD. Suppressive effects of microRNA-16 on the proliferation, invasion and metastasis of hepatocellular carcinoma cells. *Int J Mol Med.* (2015) 36:1713–9. 10.3892/ijmm.2015.2379 26499886

[70] Slaby O Redova M Poprach A Nekvindova J Iliev R Radova L Identification of MicroRNAs associated with early relapse after nephrectomy in renal cell carcinoma patients. *Genes Chromosomes Cancer.* (2012) 51:707–16. 10.1002/gcc.21957 22492545

[71] Yang X Chen BB Zhang MH Wang XR. MicroRNA-126 inhibits the proliferation of lung cancer cell line A549. *Asian Pac J Trop Med.* (2015) 8:239–42. 10.1016/S1995-7645(14)60323-0 25902169

[72] Liu B Peng XC Zheng XL Wang J Qin YW. MiR-126 restoration down-regulate VEGF and inhibit the growth of lung cancer cell lines in vitro and in vivo. *Lung Cancer.* (2009) 66:169–75. 10.1016/j.lungcan.2009.01.010 19223090

[73] Wang J Tian X Han R Zhang X Wang X Shen H Downregulation of miR-486-5p contributes to tumor progression and metastasis by targeting protumorigenic ARHGAP5 in lung cancer. *Oncogene.* (2014) 33:1181–9. 10.1038/onc.2013.42 23474761 PMC3883922

[74] Santarelli L Staffolani S Strafella E Nocchi L Manzella N Grossi P Combined circulating epigenetic markers to improve mesothelin performance in the diagnosis of malignant mesothelioma. *Lung Cancer.* (2015) 90:457–64. 10.1016/j.lungcan.2015.09.021 26431916

[75] Birnie KA Prele CM Musk AWB de Klerk N Lee YCG Fitzgerald D MicroRNA signatures in malignant pleural mesothelioma effusions. *Dis Markers.* (2019) 2019:8628612. 10.1155/2019/8628612 31481984 PMC6701424

[76] De Santi C Melaiu O Bonotti A Cascione L Di Leva G Foddis R Deregulation of miRNAs in malignant pleural mesothelioma is associated with prognosis and suggests an alteration of cell metabolism. *Sci Rep.* (2017) 7:3140. 10.1038/s41598-017-02694-0 28600498 PMC5466648

[77] Jenuwein T Allis CD. Translating the histone code. *Science.* (2001) 293:1074–80. 10.1126/science.1063127 11498575

[78] Sage AP Martinez VD Minatel BC Pewarchuk ME Marshall EA MacAulay GM Genomics and epigenetics of malignant mesothelioma. *High Throughput.* (2018) 7:20. 10.3390/ht7030020 30060501 PMC6163664

[79] Chen Z Grzybowski AT Ruthenburg AJ. Traceless semisynthesis of a set of histone 3 species bearing specific lysine methylation marks. *Chembiochem.* (2014) 15:2071–5. 10.1002/cbic.201402313 25155436 PMC4415702

[80] Crisanti MC Wallace AF Kapoor V Vandermeers F Dowling ML Pereira LP The HDAC inhibitor panobinostat (LBH589) inhibits mesothelioma and lung cancer cells in vitro and in vivo with particular efficacy for small cell lung cancer. *Mol Cancer Ther.* (2009) 8:2221–31. 10.1158/1535-7163.MCT-09-0138 19671764 PMC3605895

[81] Paik PK Krug LM. Histone deacetylase inhibitors in malignant pleural mesothelioma: preclinical rationale and clinical trials. *J Thorac Oncol.* (2010) 5:275–9. 10.1097/JTO.0b013e3181c5e366 20035240 PMC4052955

[82] Shao Y Lu J Cheng C Cui L Zhang G Huang B. Reversible histone acetylation involved in transcriptional regulation of WT1 gene. *Acta Biochim Biophys Sin (Shanghai).* (2007) 39:931–8. 10.1111/j.1745-7270.2007.00363.x 18064385

[83] Panda AC Grammatikakis I Munk R Gorospe M Abdelmohsen K. Emerging roles and context of circular RNAs. *Wiley Interdiscip Rev RNA.* (2017) 8:1386. 10.1002/wrna.1386 27612318 PMC5315638

[84] Jeck WR Sharpless NE. Detecting and characterizing circular RNAs. *Nat Biotechnol.* (2014) 32:453–61. 10.1038/nbt.2890 24811520 PMC4121655

[85] Hanahan D Weinberg RA. Hallmarks of cancer: the next generation. *Cell.* (2011) 144:646–74. 10.1016/j.cell.2011.02.013 21376230

[86] Chen LL. The biogenesis and emerging roles of circular RNAs. *Nat Rev Mol Cell Biol.* (2016) 17:205–11. 10.1038/nrm.2015.32 26908011

[87] Zhang Y Xue W Li X Zhang J Chen S Zhang JL The biogenesis of nascent circular RNAs. *Cell Rep.* (2016) 15:611–24. 10.1016/j.celrep.2016.03.058 27068474

[88] Jeck WR Sorrentino JA Wang K Slevin MK Burd CE Liu J Circular RNAs are abundant, conserved, and associated with ALU repeats. *RNA.* (2013) 19:141–57. 10.1261/rna.035667.112 23249747 PMC3543092

[89] Guo JU Agarwal V Guo H Bartel DP. Expanded identification and characterization of mammalian circular RNAs. *Genome Biol.* (2014) 15:409. 10.1186/s13059-014-0409-z 25070500 PMC4165365

[90] Pillai RS. MicroRNA function: multiple mechanisms for a tiny RNA? *RNA.* (2005) 11:1753–61. 10.1261/rna.2248605 16314451 PMC1370863

[91] Haque S Harries LW. Circular RNAs (circRNAs) in health and disease. *Genes (Basel)* (2017) 8:353. 10.3390/genes8120353 29182528 PMC5748671

[92] Hansen TB Jensen TI Clausen BH Bramsen JB Finsen B Damgaard CK Natural RNA circles function as efficient microRNA sponges. *Nature.* (2013) 495:384–8. 10.1038/nature11993 23446346

[93] Xu Z Yan Y Zeng S Dai S Chen X Wei J Circular RNAs: clinical relevance in cancer. *Oncotarget.* (2018) 9:1444–60. 10.18632/oncotarget.22846 29416705 PMC5787450

[94] Li Z Huang C Bao C Chen L Lin M Wang X Exon-intron circular RNAs regulate transcription in the nucleus. *Nat Struct Mol Biol.* (2015) 22:256–64. 10.1038/nsmb.2959 25664725

[95] Veno MT Hansen TB Veno ST Clausen BH Grebing M Finsen B Spatio-temporal regulation of circular RNA expression during porcine embryonic brain development. *Genome Biol.* (2015) 16:245. 10.1186/s13059-015-0801-3 26541409 PMC4635978

[96] Li Z Huang C Bao C Chen L Lin M Wang X Corrigendum: exon-intron circular RNAs regulate transcription in the nucleus. *Nat Struct Mol Biol.* (2017) 24:194. 10.1038/nsmb0217-194a 28170000

[97] Weng W Wei Q Toden S Yoshida K Nagasaka T Fujiwara T Circular RNA ciRS-7-A promising prognostic biomarker and a potential therapeutic target in colorectal cancer. *Clin Cancer Res.* (2017) 23:3918–28. 10.1158/1078-0432.CCR-16-2541 28174233 PMC5511556

[98] Winata P Cheng YY Williams M Lin RCY Reid G. Circular RNAs as novel biomarkers for detection of malignant pleural mesothelioma. *Asia Paci J Clin Oncol.* (2018) 14:82–3.

[99] Glazar P Papavasileiou P Rajewsky N. circBase: a database for circular RNAs. *RNA.* (2014) 20:1666–70. 10.1261/rna.043687.113 25234927 PMC4201819

[100] Dudekula DB Panda AC Grammatikakis I De S Abdelmohsen K Gorospe M. CircInteractome: a web tool for exploring circular RNAs and their interacting proteins and microRNAs. *RNA Biol.* (2016) 13:34–42. 10.1080/15476286.2015.1128065 26669964 PMC4829301

[101] Panda AC. Circular RNAs Act as miRNA Sponges. *Adv Exp Med Biol.* (2018) 1087:67–79. 10.1007/978-981-13-1426-1_6 30259358

[102] Abdelmohsen K Panda AC De S Grammatikakis I Kim J Ding J Circular RNAs in monkey muscle: age-dependent changes. *Aging (Albany NY).* (2015) 7:903–10. 10.18632/aging.100834 26546448 PMC4694061

[103] Abdelmohsen K Panda AC Munk R Grammatikakis I Dudekula DB De S Identification of HuR target circular RNAs uncovers suppression of PABPN1 translation by CircPABPN1. *RNA Biol.* (2017) 14:361–9. 10.1080/15476286.2017.1279788 28080204 PMC5367248

[104] Panda AC De S Grammatikakis I Munk R Yang X Piao Y High-purity circular RNA isolation method (RPAD) reveals vast collection of intronic circRNAs. *Nucleic Acids Res.* (2017) 45:e116. 10.1093/nar/gkx297 28444238 PMC5499592

